# A High-Resolution Time Reversal Method for Target Localization in Reverberant Environments

**DOI:** 10.3390/s24103196

**Published:** 2024-05-17

**Authors:** Huiying Ma, Tao Shang, Gufeng Li, Zhaokun Li

**Affiliations:** 1 State Key Laboratory of Integrated Service Network, Xidian University, Xi’an 710071, China; tshang@xidian.edu.cn (T.S.); ligufeng@xidian.edu.cn (G.L.); zhkli@xidian.edu.cn (Z.L.); 2 Collaborative Innovation Center of Information Sensing and Understanding, Xidian University, Xi’an 710071, China

**Keywords:** broadband low frequency, target localization, time reversal, sparse Bayesian learning, reverberant environments

## Abstract

Reverberation in real environments is an important factor affecting the high resolution of target sound source localization (SSL) methods. Broadband low-frequency signals are common in real environments. This study focuses on the localization of this type of signal in reverberant environments. Because the time reversal (TR) method can overcome multipath effects and realize adaptive focusing, it is particularly suitable for SSL in a reverberant environment. On the basis of the significant advantages of the sparse Bayesian learning algorithm in the estimation of wave direction, a novel SSL is proposed in reverberant environments. First, the sound propagation model in a reverberant environment is studied and the TR focusing signal is obtained. We then use the sparse Bayesian framework to locate the broadband low-frequency sound source. To validate the effectiveness of the proposed method for broadband low-frequency targeting in a reverberant environment, simulations and real data experiments were performed. The localization performance under different bandwidths, different numbers of microphones, signal-to-noise ratios, reverberation times, and off-grid conditions was studied in the simulation experiments. The practical experiment was conducted in a reverberation chamber. Simulation and experimental results indicate that the proposed method can achieve satisfactory spatial resolution in reverberant environments and is robust.

## 1. Introduction

Broadband low-frequency target sound source localization (SSL) is a long-standing and widely researched topic in reverberant environments [1,2]. Thus far, this remains a highly challenging issue. Reverberation generally occurs in enclosed spaces, including indoor spaces, cabins, and car interiors. SSL has numerous practical applications, such as in speech enhancement, noise control, human–robot interaction, and smart homes. This study mainly focuses on rectangular indoor rooms.

The traditional SSL method is based on the signal/channel model and signal processing and has made significant progress over the years. Currently, mainstream SSL methods include beamforming [3], time delay of arrival [4], and high-resolution spectrum estimation [5]. All of these methods achieved good localization performance in free space. However, a common problem is that the physical model assumed in these methods only considers direct sound, requires the sound source to be stable and incoherent, and has poor robustness in an environment of low signal-to-noise ratio (SNR) and reverberation. Moreover, the spatial resolution is limited, particularly at low frequencies [6].

SSL in reverberant environments is challenging because of the multipath nature of the received signals. This effect degrades SSL performance. Thus, it is important to develop SSL methods that are robust to reverberation [7]. The time reversal (TR) method derived from optical phase conjugation has attracted the attention of researchers because it can overcome the multipath effect and realize adaptive focusing without a large array [8]. The TR technique was first applied in the field of ultrasound and then gradually extended to medical diagnosis, seismology, underwater acoustics, etc. In recent years, it has been introduced in the field of indoor SSL. Fink et al. [9,10,11,12] demonstrated that the TR method can achieve SSL in reverberant environments in both time and space, but the focal spot size obtained by the TR method is larger than λ/2 (λ is a wavelength) for low-frequency sound sources. Draeger et al. [13,14] achieved and demonstrated, for the first time, the effectiveness of SSL in a reverberant silicon cavity using the TR technique with both simulation and experimentation. The experiment adopted one microphone and a pulse signal with a center frequency of 1 MHz, and the focusing resolution reached 1.1 mm, corresponding to 1/2 λ of the signal. Fink et al. [15] systematically studied the validity of the TR method in SSL from both theoretical and experimental perspectives and demonstrated the effectiveness of the TR technique based on source–receiver reciprocity. Fink et al. [16] then applied the TR method to locate the sound source with a center frequency of 975 Hz using 20 microphones and obtained a resolution of 1/2 λ in a more common reverberant environment, which is rooms. Sprik R. [17] then used the TR method based on two microphones to perform the SSL experiment on a pulse with a center frequency of 1750 Hz, and the results demonstrate its applicability in reverberant environments. Researchers have proposed several solutions from different perspectives to the half-wavelength restriction. Fink et al. [18] proposed the concept of an acoustic sink to break through the λ/2 limitation. Conti et al. [19] surrounded an audible sound source between 100 and 200 Hz with an array of microphones to realize near-field TR, and λ/20 resolution was obtained. Catheline et al. [20] studied an SSL model in the special reverberant environment of the skull based on the TR method and predicted that the model could be applied to microphone arrays. Padois et al. [21] implemented the localization of a sound source with a bandwidth of 1.5–18 kHz in a wind tunnel. However, there is no echo inside the tunnel. Mimani [22] proposed a TR method on aeroacoustic sources with 3000 Hz and 500–3000 Hz for the presence of a reflecting surface and demonstrated its effectiveness by comparing the simulation with the traditional beamforming method. Ma et al. [23] achieved 1/4 λ resolution for a pulse with a center frequency of 9000 Hz by placing subwavelength scatters. Li [24] proposed a cross-spectrum TR method corrected in the wavenumber domain. Numerical simulation and experimental results show that the spatial resolution of the proposed method can reach 1/34 λ at 100 Hz under a low SNR (0 dB) in the near field.

Currently, methods that break through the 1/2 λ limit and significantly improve spatial resolution are applied in the near field. When the sound source is several wavelengths or even far away from the microphone array, i.e., far field, although the SSL method has broken through the limit of 1/2 λ, it has not improved much. Therefore, it is crucial to seek new breakthroughs.

Signals in nature can basically or approximately be expressed in sparse form. The reconstruction theory of sparse signals has been studied in depth by Donoho [25] and Candès et al. [26]. Subsequently, Baraniuk proposed the concept of compressive sensing [27]. Recently, a sparse signal representation method based on compressive sensing (CS) was introduced to realize DOA estimation when the number of sound sources is unknown in a complex environment [28]. The signal was compressed and sampled at a low rate to considerably reduce the number of samples and improve computational efficiency [25]. In general, sound sources are considered point sources in reverberant environments, and the number of sound sources is limited. The problem of SSL in reverberant environments can be expressed by sparse approximation model [29]. Chardon [30] proposed a sparse model for a narrowband SSL in an unknown reverberant field. A spatial resolution of 3/5 λ is obtained for a sound source at 30,631 Hz. Jiang et al. [31] used CS to accurately estimate the position of the sinusoidal signal source at 16 kHz, and the accuracy was higher than that of the generalized cross-correlation method. Simard et al. [32] constructed an SSL sparse model using Green’s function. The experimental results show that for a sound source with 5000 Hz, the CS method can obtain a spatial resolution of 7/20 λ, and the localization results of the beamforming algorithm are fuzzy at this resolution. Koyama et al. [33,34] demonstrated that the problem of broadband SSL can be transformed into the problem of joint sparse support recovery in terms of frequency. Chu et al. [35] further extended the sparse constraint to achieve SSL with a bandwidth of 2400–2600 Hz and achieved a spatial resolution of 9/25 λ. Tipping et al. [36] proposed a sparse Bayesian learning (SBL) algorithm based on a correlation vector machine for a sparse linear regression model. Zhang et al. [37] demonstrated the effectiveness of the SBL method for DOA estimation of quasi-stationary signals at an off-grid position by simulation. Bai et al. [38] solved the reverberation problem using the SBL approach and demonstrated by simulation that the method is more robust than other methods in noisy environments and with grid errors. Zheng et al. [39] proposed an SBL method to resolve coherent sources by simulation in impulsive noise environments. Qin et al. [40] completed audible SSL using *L*_1_ minimization in reverberant and noisy environments. For the music signal, the array with 12 microphones has a root-mean-square error of 0.2 m.

The existing research results show that although the SSL method based on TR is suitable for reverberant environments, the localization performance of sound sources with low-frequency bands needs to be improved. There are few studies based on CS in a strong reverberant environment for wideband SSL, and the common signals in reality are broadband. Therefore, the study of broadband SSL in a reverberant environment is also a hot issue. In summary, the joint use of TR and CS theory has attracted the attention of scholars. It was first introduced into multiple-input multiple-output radar Doppler estimation and has been demonstrated to have excellent performance [41]. In the field of underwater acoustic communication, Jiang et al. [42] simulated the channel as a sparse set consisting of constant and slowly time-varying supports to derive the Kalman Filtered CS channel estimation algorithm for TR communication under the framework of dynamic CS. Experimental results demonstrate the validity of the algorithm. SBL is the most commonly used signal reconstruction method, which employs a grid model to construct an overcomplete dictionary and then uses the expectation maximization (EM) principle to update parameters iteratively [43]; it has better performance and robustness than conventional SSL methods under less snaps and low SNR [44].

Scholars have used TR and SBL methods to conduct localization research on broadband signals, but they either focus on the high-frequency range or the localization results in the low-frequency range are not extremely satisfactory. Currently, there is a lack of systematic and comprehensive research on broadband low-frequency SSL in reverberation environments. Therefore, we propose a novel SSL method based on TR and SBL, which is represented as TR–SBL, to overcome the limitations of poor spatial resolution in a reverberant environment and low SNR. The process of TR and retransmission is carried out on the computer; thus, the method is passive localization. The study is organized as follows. Section 2 discusses the SSL model in reverberant environments. Section 3 describes the numerical simulations performed to validate the proposed method. Section 4 reports the actual data experiments in a reverberation chamber and the analysis of the results. Finally, Section 5 presents the conclusions.

## 2. SSL Model in a Reverberant Environment

**Figure 1:**
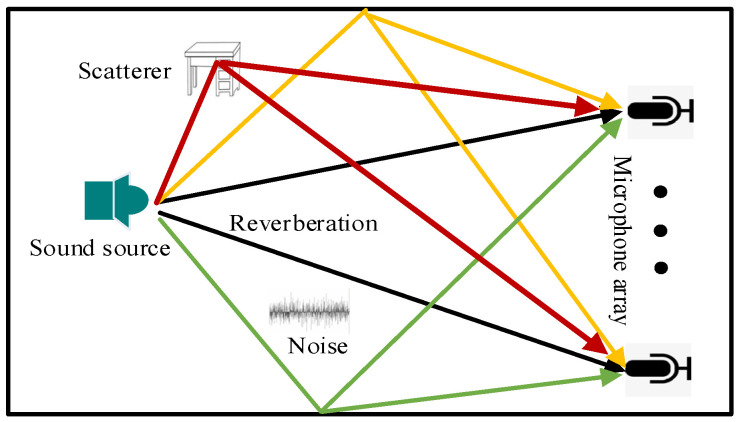
Sound propagation model in a reverberant environment.

Consider a rectangular enclosed space to simulate the sound source propagation model in a reverberant environment, as shown in Figure 1. The acoustic signal received by the *m*-th microphone is as follows:

(1) $$y_{m}\left(t\right)=x\left(t\right)⊗h_{m}\left(r_{s},r_{m},t\right)+n\left(t\right)$$

where *x*(*t*) is the sound source signal, $h_{m}\left(r_{s},r_{m},t\right)$ is the room impulse response (RIR) from the sound source position rs to the microphone position rm, ⊗ is the convolution, m is the *m*-th microphone, and *n*(*t*) is the background noise, which is generally assumed to be independent of the sound source.

As can be seen from Figure 1, RIR has not only direct sound (black line) but also reflected sound (green line, yellow line, and red line), which contains all the information of the sound field and can accurately reflect the acoustic environment (direction, distance, and sense of space). Therefore, RIR is a specific manifestation of reverberation. The reverberant environment is time-varying; obstacles, people, etc., may be different from the simulated environment, which requires strong robustness of the SSL algorithm.

When the received data are a broadband signal, the broadband processor can use the sound source information contained in multiple frequencies compared with the narrowband processor, thereby improving the SSL accuracy. The frequency domain form of Equation (1) is as follows:

(2) $$Y_{m}\left(f\right)=S\left(f\right)H_{m}\left(r_{m},r_{s},f\right)+N\left(f\right)$$

where *f* is the frequency, $Y_{m}\left(f\right)=\left[Y_{m}\left(f_{1}\right),Y_{m}\left(f_{2}\right),\ldots ,Y_{m}\left(f_{F}\right)\right]$, *F* stands for the number of frequency bands, and $S\left(f\right)=\left[S\left(f_{1}\right),S\left(f_{2}\right),\ldots ,S\left(f_{F}\right)\right]$.

The TR processing of Equation (2) can be obtained according to the frequency domain phase conjugation TR as follows:

(3) $$Y_{m}^{*}\left(f\right)=S^{*}\left(f\right)H_{m}^{*}\left(r_{m},r_{s},f\right)+N\left(f\right)$$

**Figure 2:**
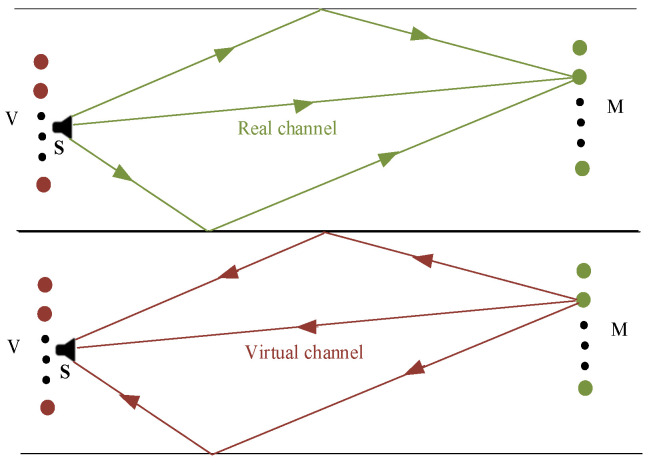
Diagram of time reversal.

The signal $Y_{m}^{*}\left(f\right)$ is reemitted back into the medium along the virtual channel $V_{m}\left(r_{vs},r_{m},f\right)$. The overall diagram of time reversal is shown in Figure 2.

As shown in Figure 2, there is another microphone array ***V*** at the initial sound source for receiving the signal propagated through the virtual channel after time reversal, and the signal received by the array ***V*** is

(4) $$\begin{array}{ll} Z_{m}\left(f\right) & =Y_{m}^{*}\left(f\right)V_{m}\left(r_{vs},r_{m},f\right)+N\left(f\right)\\ & =S^{*}\left(f\right)H_{m}^{*}\left(r_{m},r_{s},f\right)V_{m}\left(r_{vs},r_{m},f\right)+N\left(f\right) \end{array}$$

Virtual channel refers to the channel propagated by the time reversal signal. According to the spatial reciprocity theorem, the time reversal signal will be refocused on the initial sound source position along the original path, but in practical application, the process of time reversal and retransmission is carried out on the computer; thus, the method is passive localization. Since the position of the sound source is unknown, so is the virtual channel. The possible position of the sound source is defined as the area of interest, and several grids are divided in this area. It is assumed that the position of the center of each grid is the position of the sound source, and the propagation channel of the microphone array to each grid is a virtual channel, which is obtained by the image method.

Once the virtual channel matches the real channel, $H_{m}^{*}\left(r_{m},r_{s},f\right)V_{m}\left(r_{vs},r_{m},f\right)=H_{m}^{*}\left(r_{m},r_{s},f\right)H_{m}\left(r_{m},r_{s},f\right)$, it is a real, even, and positive function with the maximum space gain of each element. This is equivalent to the spatial matching filtering of each array element. Finally, the focusing energy of each array element is added to obtain the SSL result graph, as shown in Equation (5), which is similar to the beamforming technology. Beamforming achieves spatial filtering through time delay or phase matching search and compensation of each array, whereas the TR method achieves spatial filtering through TR channel matching of each array.

(5) $$R\left(f\right)=\sum_{m=1}^{M}\sum_{f=1}^{F}Z_{m}\left(f\right)Z_{m}^{*}\left(f\right)$$

**Figure 3:**
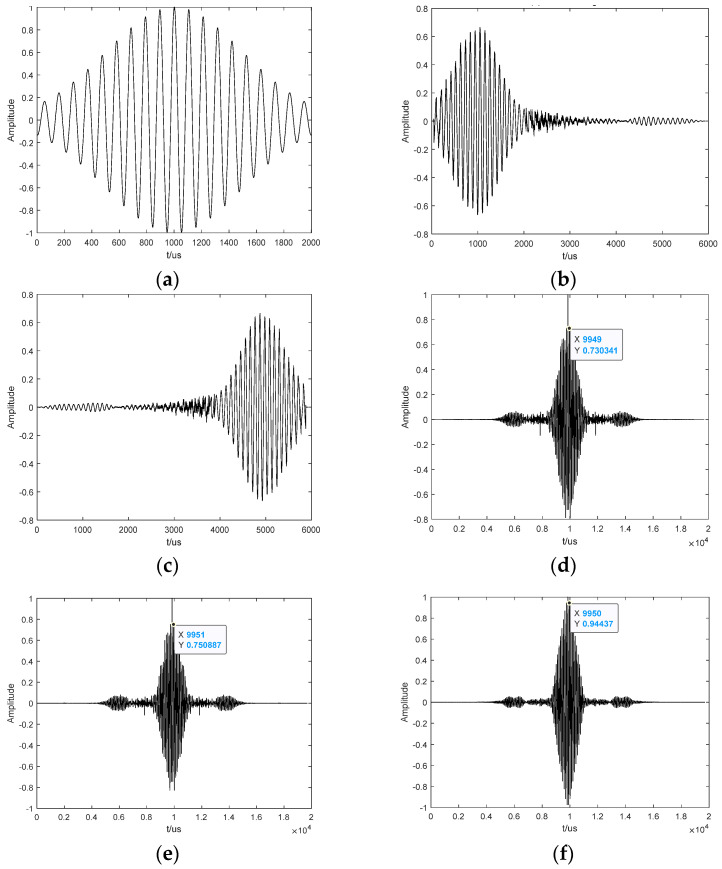
The entire process of traditional TR. (**a**) Gaussian pulse; (**b**) received signal; (**c**) TR signal; (**d**) focused signal at a non-sound-source position; (**e**) focused signal at a non-sound-source position; (**f**) focused signal at the position of the sound source.

Let us take into account a single microphone as an example of the TR process. The room size was set to 4 m × 2 m × 3 m, and the sound absorption coefficient of the six walls was 0.2. A Gaussian pulse signal with a center frequency $f_{0}=9.5\;\mathrm{kHz}$ and bandwidth of 750 Hz was used as the original sound source, and the waveform is shown in Figure 3a. The coordinates of the sound source and microphone are (2, 1, 1.5) m and (3, 1, 1.5) m, respectively. The signal received by the microphone is shown in Figure 3b. The received signal is reversed along the time axis, and a TR signal is obtained, as shown in Figure 3c. Subsequently, we follow the principle that the first arrived signal is sent later, the last arrived signal is transmitted first, and it is retransmitted back to the medium. The second received signal is processed according to Equation (5). When the signal returns along the initial path, a focused signal can be formed at the original position, as shown in Figure 3d. Suppose that the signal after the TR process does not return according to the initial path, then the return path is between (3, 1, 1.5) m, (1.9, 1, 1.5) m, and (0.05, 0.5, 1.0) m. The focused signal is shown in Figure 3e,f. It can be seen from the last three subgraphs that (1) when the virtual channel is the same as the initial channel, there is an obvious focus peak and (2) when the virtual channel is not the same as the initial channel but is extremely close to the initial channel, the focused signal will also have a peak value. However, the second peak value in Figure 3e is larger than that in Figure 3d; therefore, (3) when the virtual channel is far from the initial channel, the peak value is not obvious, and even the focus is achieved in other positions.

**Figure 4:**
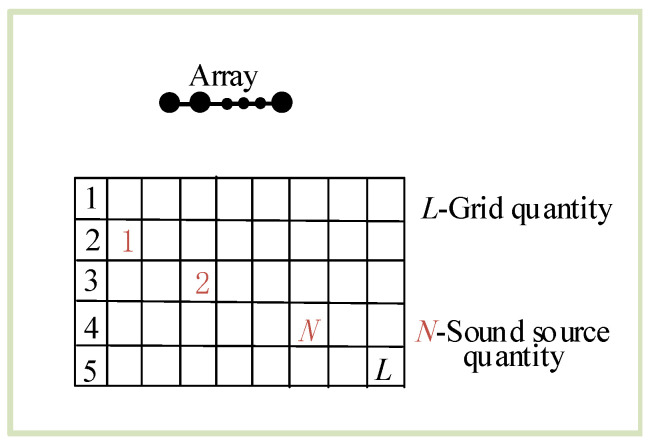
Spatial sparse representation.

To solve the problem that the sidelobe value of the TR method is relatively large, we use the SBL method for postprocessing. The area of interest in reverberant environments is divided into *L* grids, and it is assumed that there may be a sound source on each grid. There are *N* sound sources, and with L≫N, the signal satisfies the sparse hypothesis, as shown in Figure 4. The matrix formed by the RIR of all sound sources to the microphone array forms an overcomplete basis. The more grids there are, the smaller the spacing between them, the higher the localization accuracy, but the larger the corresponding calculation amount.

The frequency domains of the TR signals received by the microphone array are as follows:

(6) $$\begin{array}{ll} Z_{f} & ={\left[\begin{array}{c} S_{1}^{*}\left(f\right)\\ S_{2}^{*}\left(f\right)\\ \vdots \\ S_{L}^{*}\left(f\right) \end{array}\right]}^{T}\cdot {\left[\begin{array}{cccc} H_{11}^{*}\left(f\right) & H_{12}^{*}\left(f\right) & \cdots & H_{1L}^{*}\left(f\right)\\ H_{21}^{*}\left(f\right) & H_{22}^{*}\left(f\right) & \cdots & H_{2L}^{*}\left(f\right)\\ \vdots & \vdots & \vdots & \vdots \\ H_{M1}^{*}\left(f\right) & H_{M2}^{*}\left(f\right) & \cdots & H_{ML}^{*}\left(f\right) \end{array}\right]}^{T}\cdot \left[\begin{array}{cccc} V_{11}\left(f\right) & V_{12}\left(f\right) & \cdots & V_{1L}\left(f\right)\\ V_{21}\left(f\right) & V_{22}\left(f\right) & \cdots & V_{2L}\left(f\right)\\ \vdots & \vdots & \vdots & \vdots \\ V_{M1}\left(f\right) & V_{M2}\left(f\right) & \cdots & V_{ML}\left(f\right) \end{array}\right]+N\left(f\right)\\ & =A_{f}\cdot {\bar{S}}_{f}+N_{f} \end{array}$$

where $A_{f}={\left(H^{*}\left(f\right)\right)}^{T}\cdot V\left(f\right)$ and ${\bar{S}}_{f}={\left[S_{1}^{*}\left(f\right),S_{2}^{*}\left(f\right),\ldots ,S_{L}^{*}\left(f\right)\right]}^{T}$. The dimensions of each of these terms are as follows: $Z_{f}\in {\mathbb{C}}^{L\times 1}$, $A_{f}\in L\times L$, and ${\bar{S}}_{f}\in L\times 1$. ***A****_f_* is a dictionary composed of redundant RIR. Equation (6) is an underdetermined equation.

1. Noise model

The noise is assumed to conform to a complex normal distribution with the precision $\alpha_{0}$, so the likelihood function of Equation (6) is as follows:

(7) $$Z_{f}\left|{\bar{S}}_{f},\right.\alpha_{0}∼CN\left(A_{f}{\bar{S}}_{f},\alpha_{0}^{-1}I\right)$$

where $\alpha_{0}$ is an unknown parameter that is assumed to obey the distribution of gamma with hyperparameters a and b [45], the symbol CN denotes the complex normal distribution, and ***I*** is an identity matrix. A gamma prior with hyperparameters *a* and *b* is further imposed on $\alpha_{0}$

(8) $$p\left(\alpha_{0}\left|a,b\right.\right)=\Gamma \left(\alpha_{0}\left|a,b\right.\right)$$

2. Sparse signal model

For the sparse signal ${\bar{S}}_{f}$, we adopt a two-stage hierarchical sparse prior model to describe it:

(9) $${\bar{S}}_{f}\left|\alpha ∼CN\left(0,\Lambda^{-1}I\right)\right.$$

(10) $$p\left(\alpha \left|\varphi \right.\right)=\prod_{n=1}^{N}\Gamma \left(\alpha \left|1,\varphi \right.\right)$$

where $\Lambda =\mathrm{diag}\left(\alpha \right)$ and ${\left\{{\bar{S}}_{f}\right\}}_{f=1:F}$. According to conventional SBL, parameters *a*, *b*, and φ in Equations (8) and (10) are commonly set to be 10^−6^ [36].

According to the above analysis, the joint probability density function can be expressed as:

(11) $$p\left({\left\{{\bar{S}}_{f}\right\}}_{f=1:F},{\left\{Z_{f}\right\}}_{f=1:F},\alpha_{0},\alpha \right)=p\left({\left\{Z_{f}\right\}}_{f=1:F}\left|{\left\{{\bar{S}}_{f}\right\}}_{f=1:F}\right.,\alpha_{0}\right)p\left({\left\{{\bar{S}}_{f}\right\}}_{f=1:F}\left|\alpha \right.\right)p\left(\alpha \right)p\left(\alpha_{0}\right)$$

3. Bayesian Inference

By treating ${\bar{S}}_{f}$ as a hidden variable, whose posterior distribution is as follows:

(12) $$p\left({\bar{S}}_{f}\left|Z_{f}\right.,\alpha_{0},\alpha \right)=\prod_{n=1}^{N}CN\left({\bar{S}}_{f}\left|\mu \right.,\Sigma \right)$$

with

(13) $$\begin{array}{l} {\bar{S}}_{f}=\mu \\ \mu =\alpha_{0}\Sigma A_{f}^{H}Z_{f}\\ \Sigma_{f}={\left(\Delta +\alpha_{0}A_{f}^{H}Z_{f}\right)}^{-1} \end{array}$$

calculations of μ and $\Sigma_{f}$, need estimates of the hyper-parameters $\alpha_{0}$ and α. The maximum posterior probability is used to calculate $\alpha_{0}$ and α.

(14) $$p\left(Z_{f},\alpha_{0},\alpha \right)=p\left(\alpha_{0},\alpha \left|Z_{f}\right.\right)p\left(Z_{f}\right)$$

Each parameter in *p*(***Z****_f_*) is independent, and maximizing $p\left(\alpha_{0},\alpha \left|Z_{f}\right.\right)$ is the same as maximizing $p\left(Z_{f},\alpha_{0},\alpha \right)$.

Since $p\left({\bar{S}}_{f},\alpha_{0},\alpha \left|Z_{f}\right.\right)$ cannot be explicitly calculated, we adopt variational Bayesian inference (VBI) to approximate the posterior and infer hidden variables. The optimization problem within a distribution $q\left(\Delta \right)$ with a factorized form is assumed to approximate the true posterior, and based on Kullback–Leibler divergence (KL), true posterior $p\left(\Delta \left|z\right.\right)$ can be expressed as follows:

(15) $$\begin{array}{ll} q^{*}\left(\Delta \right) & =\underset{q\left(\Delta \right)}{\arg\;\mathrm{minKL}}\left(q\left(\Delta \right)\left|p\left(\Delta \left|z\right.\right)\right.\right)\\ & =\underset{q\left(\Delta \right)}{\arg\;\mathrm{minKL}}\left(\int q\left(\Delta \right)\log\frac{q\left(\Delta \right)}{p\left(\Delta \left|z\right.\right)}d\Delta \right) \end{array}$$

where $q^{*}\left(\Delta \right)$ stands for estimated value.

$q\left(\Delta \right)$ can be written as follows:

(16) $$q\left(\Delta \right)=q\left(\left\{{\left\{{\bar{S}}_{f}\right\}}_{f=1,\ldots ,F},\alpha ,\alpha_{0}\right\}\right)=q\left(\alpha_{0}\right)q\left(\alpha \right)\prod_{f}q\left({\bar{S}}_{f}\right)$$

On the basis of the above derivation, the optimal distribution of Equation (15) can be expressed as follows:

(17) $$\ln\;q^{*}\left(\Delta_{k}\right)=E{\left(\ln\;p\left(z_{f},\Delta \right)\right)}_{q\left(\Delta \backslash \Delta_{k}\right)}$$

where $E{\left(\ln\;p\left(z_{f},\Delta \right)\right)}_{q\left(\Delta \backslash \Delta_{k}\right)}$ denotes the expectation with respect to $q\left(\Delta \backslash \Delta_{k}\right)$, and $\Delta \backslash \Delta_{k}$ denotes the set Δ without $\Delta_{k}$.

4. Hidden variables updating

Hidden variables are updated using the VBI technique. The updating rule for each type of variable is derived from Equation (17). The updated estimations of α and $\alpha_{0}$ are as follows:

(18) $$\begin{array}{l} \alpha^{new}=\frac{c+F}{d+\sum_{f}\mathrm{diag}\left(\mu_{f}\mu_{f}^{H}+\Sigma_{f}\right)}\\ \alpha_{0}^{new}=\frac{a+MF}{b+\Sigma_{f}\left\{{\left\|z_{f}-A_{f}\mu_{f}\right\|}_{2}^{2}+\mathrm{Tr}\left(A_{f}\Sigma_{f}A_{f}^{H}\right)\right\}} \end{array}$$

where Tr is the operation of trace.

The parameters are always updated and stopped when the maximum error between two consecutive values is quite small or the number of iterations has reached the preset value. We can obtain final μ, so the positions of the sound sources can be estimated from it.

## 3. Simulations and Analysis

In this section, first, the effectiveness of the proposed TR–SBL method is verified by comparing it with the TR method and *L*_1_ minimization. Second, the SSL performance of the proposed TR–SBL method is verified under different parameter settings, including different bandwidths, a different number of microphones, SNRs, reverberation times, and off-grid conditions. These methods are implemented on the MATLAB R2022a platform installed on a computer with the following parameters: Intel (R) Core (TM) i7-13700KF CPU @3.40 GHz. The computer manufacturer is Intel, from Xi’an, China.

**Figure 5:**
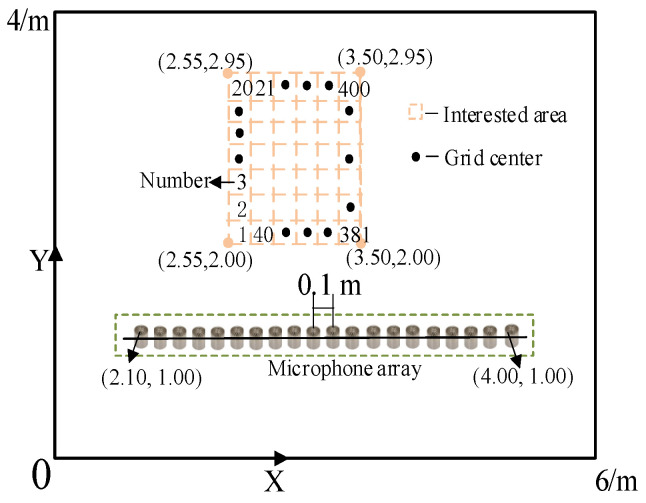
Sketch of the computational domain with the position of the mic and sound source.

First, select a rectangular room with a size of 6 m × 4 m × 3 m as the reverberant environment. The array comprises 20 omnidirectional microphones with intervals of 0.1 m. The array is distributed along the *x*-axis from 1.0 to 2.9 m, and the *y*-axis and *z*-axis coordinates are 1.0 m. We have selected the area of interest at a certain distance from the array, aiming to position it as centrally as possible within the entire room. This is performed to avoid proximity to walls or corners, which could lead to destructive interference or phase addition effects. The coordinates of the area of interest are 2.55–3.50 m on the *x*-axis, 2.0–2.95 m on the *y*-axis, and 1.0 m on the *z*-axis. The area of interest is divided into 400 grids with intervals of 0.05 m. The spatial impulse response from the center of each grid to the microphone array is simulated using the Image method. The bandwidths of the sound source are 125–250, 250–500, 500–1000, and 125–1000. The SNR is 20 dB. The coordinates of the sound source are (2.55, 2.75, 1.00) m. A sketch of the computational domain with the position of the microphone array and sound source is shown in Figure 5.

The reverberation time is usually denoted by T_60_, which is defined as when the sound field reaches a steady state and the sound source stops sounding. It takes time for the sound pressure level to decrease by 60 dB [46]. The unit is second. The reverberation time T_60_ of an ordinary room is determined by the volume *V*, surface area *S*, and sound absorption coefficient α.

(19) $$T_{60}=0.161\frac{V}{-S\log_{e}\left(1-\bar{\alpha }\right)}$$

where $\bar{\alpha }$ is the average sound absorption coefficient. In this study, we assume that the sound absorption coefficient of all walls is the same, which is α.

### 3.1. Comparison with TR and L_1_ Norm Minimization

**Figure 6:**
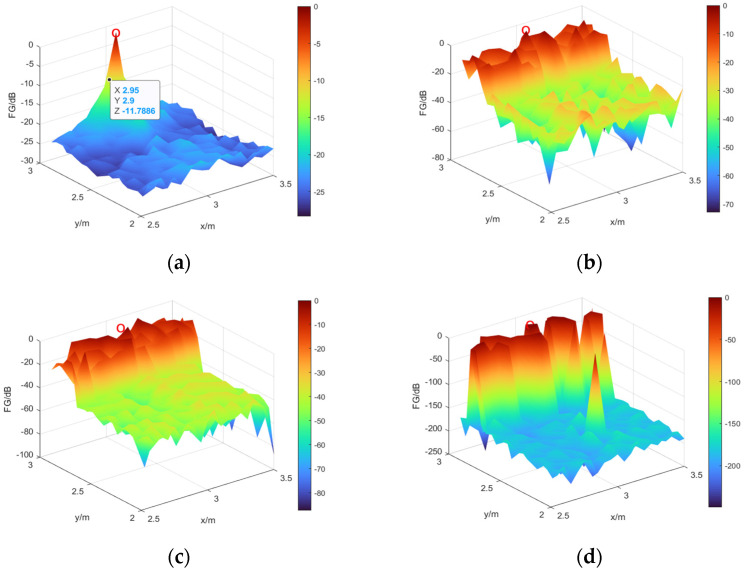

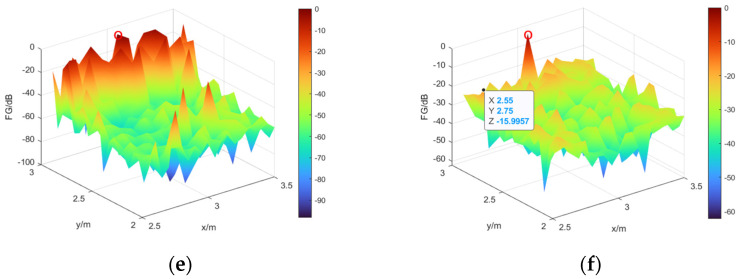
SSL results of TR method, *L*_1_ norm minimization, and TR–SBL method. (**a**) TR; (**b**) *L*_1_ norm, λ = 10^−10^; (**c**) *L*_1_ norm, λ = 10^1^; (**d**) *L*_1_ norm, λ = 10^−1^; (**e**) *L*_1_ norm, λ = 10^−8^; (**f**) TR–SBL.

To verify the effectiveness of the TR–SBL method, the conventional TR method and *L*_1_ norm minimization are compared. All algorithms are implemented on the MATLAB platform. The bandwidth of the sound source is 125–1000 Hz, and the sound source is located at (3.00, 2.90) m. The sound absorption coefficient α is 0.5. T_60_ is 153.9 ms. Figure 6 shows the SSL results obtained using the three methods. The SSL results are represented by the focusing gain *FG*:

(20) $$FG=10\log_{10}\left(\sum_{i=1}^{M}R{\left(f\right)}_{i}/R{\left(f\right)}_{i\max}\right)$$

The actual position of the sound source is indicated by a red circle. The SSL results of the TR and TR–SBL methods are shown in Figure 6a and Figure 6f, respectively, and the rest of the subfigures are SSL results of *L*_1_ norm minimization under $\lambda =10^{-10},10^{1},10^{-1},10^{-8}$.

As can be seen from Figure 6, the SSL results obtained using different methods vary greatly. In terms of the obtained position, both the traditional TR method and the TR–SBL method can obtain accurate positions. The difference between the two methods is mainly the value of the second peak and the remaining sidelobe. The second peak value of the proposed TR–SBL method is 26% lower than that of the TR method, and the performance is greatly improved. The SSL position obtained from *L*_1_ norm is greatly affected by the value of parameter λ. When the parameter λ selection is appropriate, such as 10−8, an accurate SSL position can be obtained, but the sidelobe value is quite large. When the parameter λ selection is not appropriate, such as 10^−10^, 10^1^, and 10^−1^, the positions of the sound source obtained by *L*_1_ norm are all (3.05 and 2.85), and the corresponding SSL error is 0.07 m. The selection of parameter λ is irregular; thus, we can only keep trying. From the peak value perspective, the proposed TR–SBL method obtains the highest performance and the lowest sidelobe value.

We take into account the root-mean-square error (RMSE) as an evaluation criterion for further analysis, which is defined as follows:

(21) $$\mathrm{RMSE}=\sqrt{\frac{1}{N}\sum_{i=1}^{N}{\left(r_{s,i}-r_{a,i}\right)}^{2}}$$

where *N* is the number of Monte Carlo experiments, $r_{s,i}=(x_{s,i},y_{s,i},z_{s,i})$ is the position obtained by simulation calculation, and $r_{a,i}=(x_{a,i},y_{a,i},z_{a,i})$ is the actual position.

Subsequently, we compare the RMSEs of the three algorithms at different SNRs, mainly in the case of low SNR. Twenty Monte Carlo experiments were performed for each SNR, and $\lambda =10^{-8}$; the results are shown in Table 1.

It can be seen from the results of the RMSEs that under the same SNR, the traditional TR method has the largest RMSE, whereas the proposed TR–SBL method has the smallest; all are 0 cm. In the case of low SNR, −30 dB, the result of the TR method is 0.75 m, and that of the *L*_1_ norm method is 0.58 m. With an increase in SNR, the RMSE of the two traditional methods is reduced. When the SNR is greater than −20, the RMSE of the TR method decreases to 0. The RMSE of the *L*_1_ norm method decreases with an increase in the SNR, but it does not decrease to 0.

As is well known, the more grids there are, the greater the computational workload. In practical applications, the time-consuming nature of the algorithm is a factor that cannot be ignored, which determines whether it is suitable for engineering applications. Therefore, Table 2 compares the elapsed time of different methods under the same conditions and different grid numbers. The SNR is 20 dB and λ is 10^−8^.

As can be seen from Table 2, as the number of grids increases, so does the corresponding elapsed time; however, the increasing range is not significant. When the number of grids is the same, the TR method has the optimal performance, that is, the shortest elapsed time; *L*_1_ norm minimization has the worst performance, that is, the longest elapsed time; and the elapsed time required by the proposed method falls between the two.

### 3.2. Analysis under Different Parameter Settings

We investigated the SSL performance of TR–SBL methods under different bandwidths, SNRs, reverberation times, and off-grid conditions in this section. In addition to the RMSE mentioned above, we added the SSL probability of accurate localization index to evaluate the performance, which is defined as

(22) $$\Theta =\frac{C\left(\mathrm{Correct}\;\mathrm{localization}\;\mathrm{number}\right)}{M\left(\mathrm{Monte}\;\mathrm{Carlo}\;\mathrm{experiments}\;\mathrm{number}\right)}$$

#### 3.2.1. Different Bandwidths

The types of broadband signals in a reverberant environment are not fixed, but they are basically low- and medium-frequency signals. Therefore, we conducted research on SSL performance at different bandwidths to verify its robustness.

**Figure 7:**
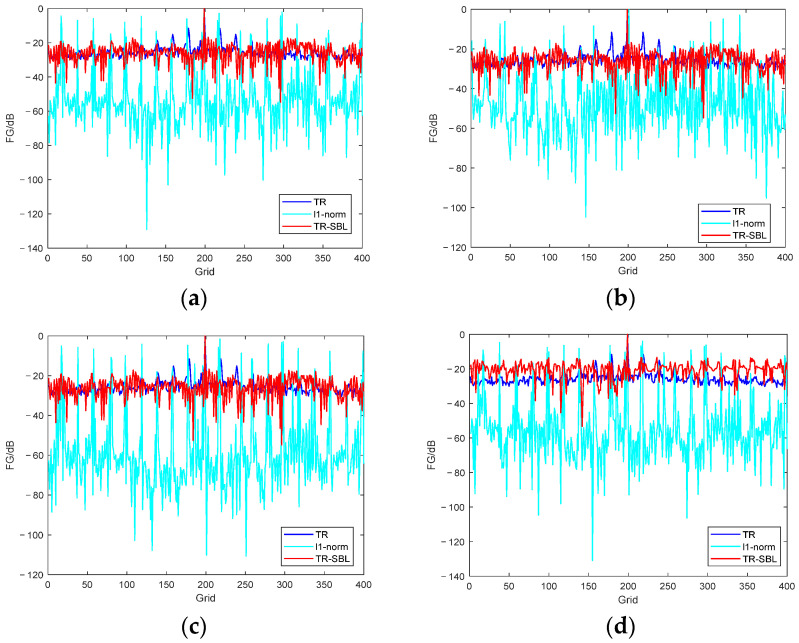
SSL results of the three methods under different bandwidths. (**a**) 125–250 Hz; (**b**) 250–500 Hz; (**c**) 500–1000 Hz; (**d**) 125–1000 Hz.

Broadband signals with bandwidths of 125–250, 250–500, 500–1000, and 125–1000 Hz were selected. Figure 7 shows the SSL results of the three methods under different bandwidths.

As can be seen from Figure 7, that the maximum and minimum sidelobe values come from the *L*_1_ norm method for the same sound source. Although the L1 norm can obtain the correct position, the sidelobe values are quite large, close to 0 dB. The maximum sidelobe value obtained by the TR method is approximately −10 dB, and the maximum sidelobe value obtained by the proposed TR–SBL method is approximately −20 dB. The overall sidelobe value of the proposed method is also lower than that of the TR method. In general, the proposed method has better SSL performance.

**Figure 8:**
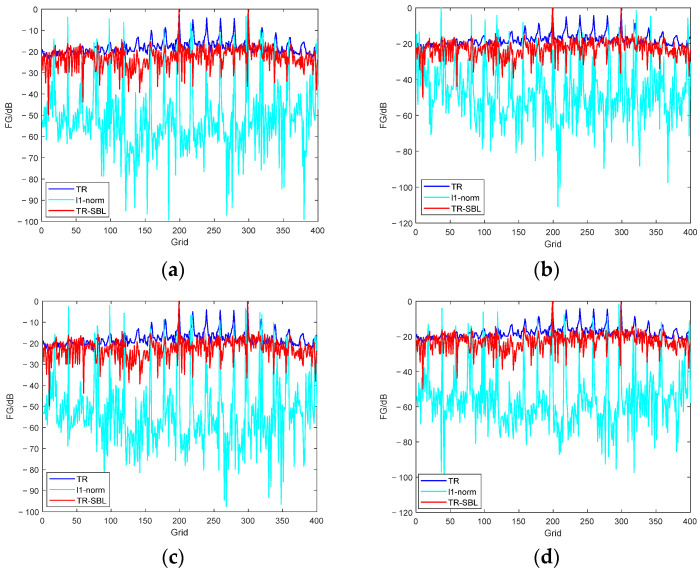
Comparison of SSL results of different methods for dual sound sources. (**a**) 125–250 Hz; (**b**) 250–500 Hz; (**c**) 500–1000 Hz; (**d**) 125–1000 Hz.

We further investigated the performance of the method when there are two sound sources in space, and the results are shown in Figure 8. Among the methods, the λ value of the *L*_1_ norm method is 10^−8^. The actual position coordinates of the two sound sources are (3.00, 2.90, 1.00) and (3.25, 2.90, 1.00) m, respectively.

As can be seen from Figure 8 that the proposed TR–SBL method can accurately find the position of dual sound sources, and the sidelobe value is significantly lower than that of the TR method. The maximum sidelobe difference between the two methods is approximately 10 dB. The sidelobe value of the *L*_1_ norm method is too high, resulting in an unclear display of the sound source position.

#### 3.2.2. Different Numbers of Microphones

**Figure 9:**
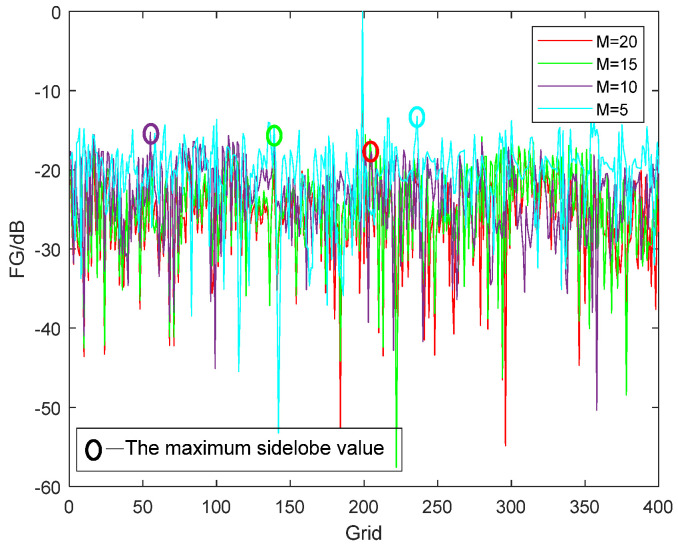
SSL results with different numbers of microphones.

Generally, the more microphones in the array element, the greater the spatial gain and the better the SSL result. However, in engineering applications, what we seek is to obtain better results with as few microphones as possible. Therefore, we further investigate the robustness of the proposed method with different numbers of microphones. We take into account the number of microphones as M = 20, 15, 10, and 5; the SNR is 20 dB; and the sound source is 125–250 Hz. The SSL results are shown in Figure 9.

Figure 9 shows that even if the number of microphones is reduced to five, the method can obtain the exact position of the sound source, which demonstrates that it has high robustness. The SSL results of different microphones are different in the sidelobe value. The smaller the number of microphones is, the higher the sidelobe value is.

#### 3.2.3. Different SNRs

**Figure 10:**
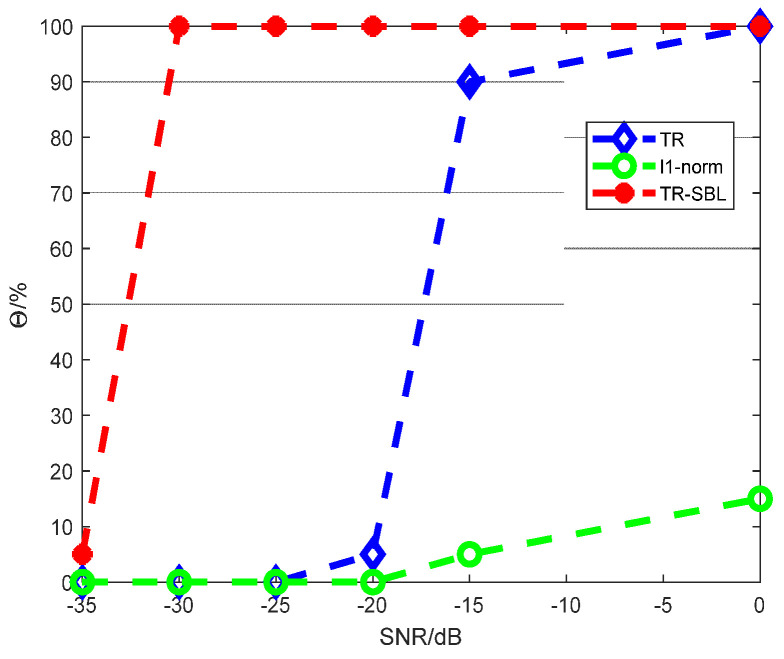
SSL probability of accurate localization Θ under different SNRs.

The noise in reverberant environments is also an important factor affecting the high spatial resolution of the SSL method. Therefore, the SSL performance of the proposed TR–SBL method is studied under different SNRs; −35, −30, −20, −15, and 0 dB are selected. The SSL probability of accurate localization Θ is used as an evaluation index. Twenty Monte Carlo experiments are conducted, and the SSL results are illustrated in Figure 10.

As shown in Figure 10, in the case of an extremely low SNR of −35 dB, although the SSL Θ of the proposed TR–SBL method is only 5%, the other two methods have a lower Θ, which is 0%. When the SNR rises to −30 dB, the proposed method can obtain 100% SSL Θ, whereas the Θ of the other two methods is 0%. When the SNR is 0 dB, the SSL Θ of the TR method reaches 100%. The accuracy of the *L*_1_ norm method has been extremely low, and the highest is only 15%. In general, the proposed method can obtain a high Θ at a lower SNR.

#### 3.2.4. Different Reverberation Times

The localization performance of the algorithm is greatly affected by reverberation. The reverberation time is generally determined by the sound absorption coefficient α and the room size. Rooms include walls, ceilings, and floors, and each of them may have different sound absorption coefficients. In the present study, we assume that each wall has the same sound absorption coefficient α.

We take into account 0.2, 0.4, and 0.6 as sound absorption coefficients for research, and their corresponding reverberation times are 0.3330, 0.1455, and 0.0811 s, respectively. Four kinds of broadband SSL simulation experiments are completed in the three reverberant environments. After 20 Monte Carlo experiments on 4 broadband sound sources, the final probability of accurate localization Θ is 100%, indicating that the proposed TR–SBL method is robust in a reverberant environment.

We also reviewed several SSL methods that have been applied in typical environments with noise and reverberation over the past two years, with some of them presented as examples: Jiajun Yan et al. [47] fed the data extracted from a time-domain feature-vector based on the sample covariance of microphone array data. The simulation results showed that the SSL error was 0.2 m when the SNR was 10 dB and the reverberation time T_60_ was 0.1 s. Guillermo et al. [48] proposed exploiting spatial diversity as an alternative approach to achieve robustness against reverberation. When the reverberation time was 0.2 s, the SSL error of 0.3 m was obtained. There is no mention of the SNR in this study. Compared with these two methods, the proposed method in this paper has satisfactory performance.

#### 3.2.5. Off-Grid Conditions

**Figure 11:**
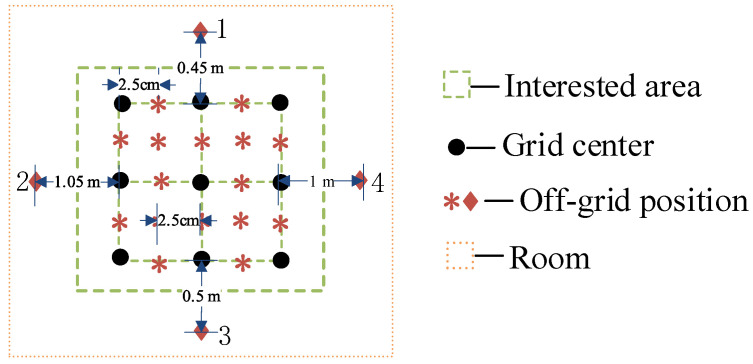
Diagrammatic sketch of different sound source positions.

In practical engineering applications, the position of the sound source is random and uncertain, meaning that the sound source may appear at any position in the room, but if each position is searched once, the computational complexity is huge. Therefore, we need to study the robustness of the proposed TR–SBL method when the sound source is not in the center of the grid, which is an off-grid condition. Figure 11 provides a detailed description of the position of off-grid conditions in different situations. Positions that are not in the center of the grid are generally divided into two categories: (1) outside the area of interest, as shown by the ♦ in Figure 11, and (2) within the area of interest, as shown by the ∗ in Figure 11.

**Figure 12:**
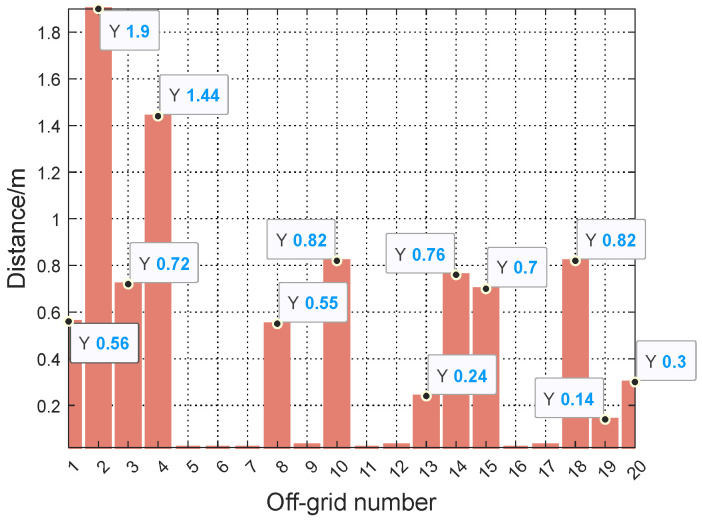
SSL results for off-grid conditions.

The sound source bandwidth is 125–1000 Hz, the sound absorption coefficient α is 0.4, and the SNR is 20 dB. The distance from the position obtained by the TR–SBL algorithm to the actual position is used to represent the SSL result, as shown in Figure 12. Points 2 and 4 located outside the area of interest have the largest SSL results from the actual position: 1.9 and 1.44 m, respectively. The SSL results for points 1 and 3 are 0.56 and 0.72 m, respectively. For the points within the area of interest, the distance of points 8, 10, 14, 15, and 18 obtained by the TR–SBL algorithm is more than 0.5 m, and the farthest distance is 0.82 m. The distance of points 8, 10, 14, 15, and 18 is more than 0.5 m; the farthest distance is 0.82 m; and the distance of points 13, 19, and 20 is between 0.1 and 0.3 m. The remaining eight points are less than 0.03 m, which is basically negligible. In general, points outside the area of interest have greater distances than points inside the area of interest.

## 4. Real Data Experiments

To demonstrate the effectiveness of the proposed TR–SBL method, real data experiments are conducted in a real rectangular room with strong reverberation, i.e., a reverberation chamber. The corresponding details and results of these experiments are given below.

**Figure 13:**
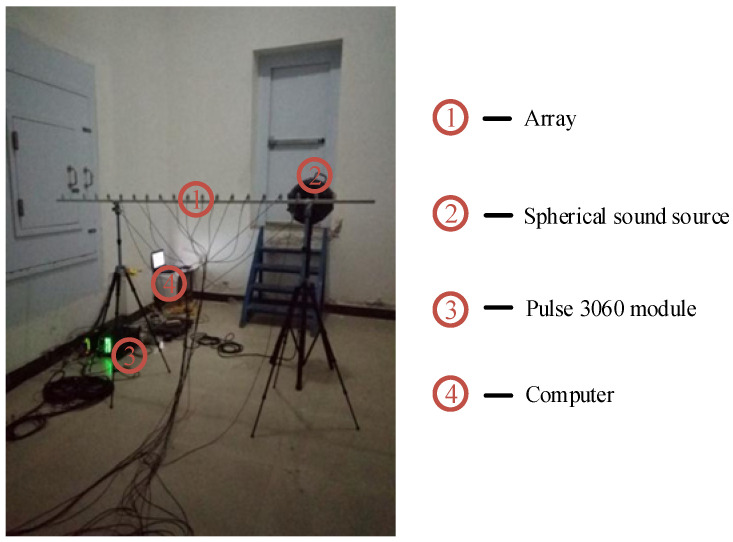
Photograph of equipment in a reverberation chamber.

The sound sources are recorded by a uniform linear array consisting of 15 omnidirectional microphones. The microphone type is 4938 (Brüel & Kjær, Virum, Denmark), and the frequency response range is 4 Hz to 70 kHz. A photograph of the equipment in a reverberation chamber is shown in Figure 13. The spherical sound source serves as the sounding device with a diameter of 0.5 m and the Pulse 3060 module as the receiving device (both from Brüel & Kjær).

**Figure 14:**
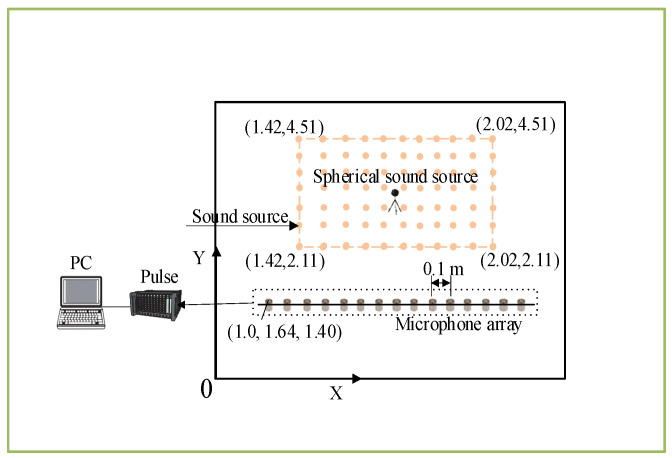
Schematic diagram of the array and sound source coordinates.

The coordinates of each device and sound source are shown in Figure 14. The reverberation chamber size is 3.24 m × 5.73 m × 4.80 m. The array is arranged along 1.0–2.4 m with a spacing of 0.1 m along the *x*-axis and 1.64 m along the *y*-axis. The *z*-axis coordinate, i.e., height, is 1.40 m. There are 15 microphones in total. The computer and pulse are placed in the corner, with negligible coordinates. The orange square represents the designated area of interest, has a height of 1.4 m, and is at the same level as the array. The range of the area of interest in the *x*-axis direction is 1.42–2.02 m, and the *y*-axis is 2.11–4.51 m. The area is divided into grids at intervals of 0.1 m, totaling 175 grids, which are represented by the orange circle. We collect signals from six positions inside the area of interest with coordinates: (2.02, 2.11, 1.40), (1.42, 2.11, 1.40), (2.02, 3.31, 1.40), (1.42, 3.31, 1.40), (2.02, 4.51, 1.40), and (1.42, 4.51, 1.40) m. We select signals with a bandwidth of 125–250, 250–500, 500–1000, and 125–1000 Hz as the sound source. Considering that the SNR in real-world environments is often much higher than in ideal conditions, the SNR is set to 20 dB.

**Figure 15:**
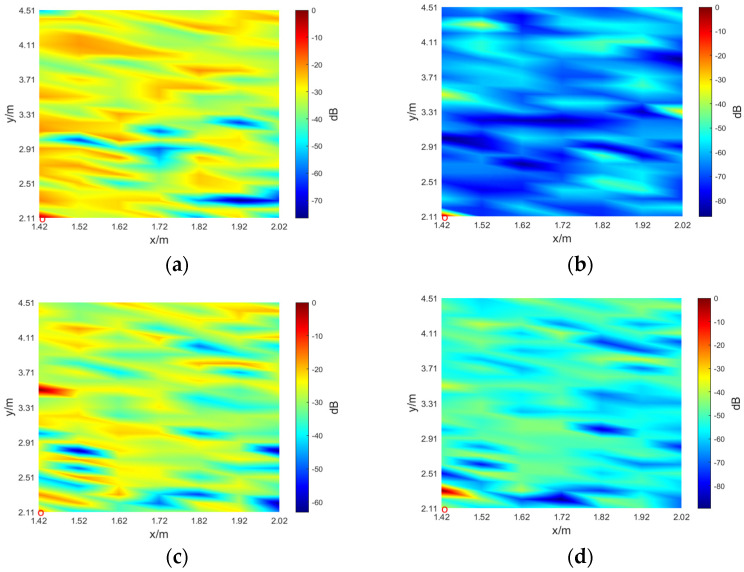

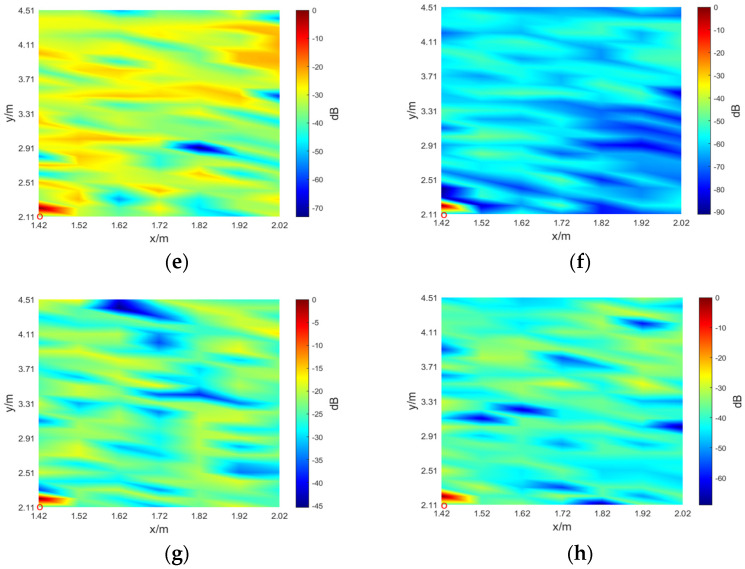
SSL results of the TR–SBL method. (**a**) Result of non-whitening for 125–250 Hz signal; (**b**) result of whitening for 125–250 Hz signal; (**c**) result of non-whitening for 250–500 Hz signal; (**d**) result of whitening for 250–500 Hz signal; (**e**) result of non-whitening for 500–1000 Hz signal; (**f**) result of whitening for 500–1000 Hz signal; (**g**) result of non-whitening for 125–1000 Hz signal; (**h**) result of whitening for 125–1000 Hz signal.

The SSL results obtained by processing signals ranging from 125 to 250 Hz are shown in Figure 15a, which are extremely blurry. To obtain clearer localization results, the received signals are whitened.

(23) $$\hat{Z}\left(\omega \right)=Z\left(\omega \right)/\left|Z\left(\omega \right)\right|$$

Figure 15 shows the SSL results of different sound sources before (figures on the left) and after (figures on the right) whitening. The red circle represents the true position of the sound source.

As can be seen from Figure 15, the SSL results of the proposed method for the nonwhitening signals are far from ideal. In Figure 15a, the experimental result of the 125–250 Hz signal is too fuzzy to find the true position of the sound source. After whitening, Figure 15b is much clearer, and the position of the sound source can be directly seen. The nonwhitening experimental results of the 250–500 Hz signal are far from the actual position, which is 1.4 m. However, after whitening, the spatial resolution can reach 0.2 m. The spatial resolution of the sound source at 500–1000 and 125–1000 Hz is the same, which is 0.1 m, regardless of whether it is nonwhitening or whitening, and the experimental results of the whitened signal are clearer and the sidelobe value is lower.

**Figure 16:**
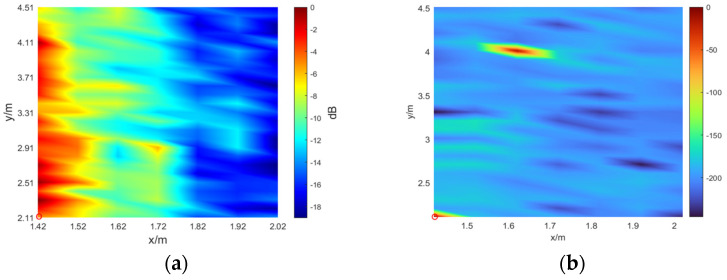
The SSL results of the TR method and the *L*_1_ norm method. (**a**) TR; (**b**) *L*_1_ norm.

Subsequently, we further compared the SSL performance of the three methods. Taking into account the sound source of 250–500 Hz as an example, we demonstrate the results of the TR method and the *L*_1_ norm method, where λ is 1, as shown in Figure 16.

From Figure 16, it can be seen that there is a certain deviation in the position of the sound source identified by the TR method, with a spatial resolution of 0.2 m. The *L*_1_ norm method can obtain the accurate position of the sound source. It is obvious from Figure 16 that the common limitation of these two methods is that the sidelobe value is extremely large, meaning that the SSL result graph cannot be clearly displayed.

We process the collected signals from the six positions and take into account the RMSE as the index to evaluate the SSL performance of the three methods, as shown in Table 3.

Overall, the RMSE of the proposed TR–SBL method is the smallest, and the RMSE of the TR method is the largest. At the low-frequency band of 125–250 Hz, the RMSE of the TR method is 0.50 m, corresponding to 7/25 λ, whereas the other two methods are both 0.37 m, corresponding to 1/5 λ, an improvement of 26%. For the frequency band of 250–500 Hz, the RMSE of the proposed method is reduced to 0.17 m, corresponding to 19/100 λ, the TR method is increased, and the *L*_1_ norm method has a slight change, which can be ignored. For the frequency band of 500–1000 Hz, the proposed method further reduces the RMSE to 0.10 m, corresponding to 11/50 λ, whereas the TR method is reduced to 0.59 m, corresponding to 1.3 λ, and the L1 norm method has slight changes. For the frequency band of 125–1000 Hz, the RMSE of the proposed method and the L1 norm method are both 0.10 m, corresponding to 9/50 λ, whereas the TR method has an RMSE of 0.64 m, corresponding to 1.3 λ, which is significant.

## 5. Conclusions

This study reformulates the SSL problem under TR and SBL, which aims to achieve high performance and strong robustness at broadband low-frequency sound sources in reverberant environments. The proposed TR–SBL method fully utilizes the antireverberation properties of the TR method and the super-resolution characteristics of the SBL method. By comparing it with the traditional TR method and *L*_1_ norm minimization, it has been demonstrated that this method performs better and has stronger robustness. Simulation results indicate that the proposed TR–SBL method obtains an accurate position for a broadband low-frequency sound source successfully in a low SNR and reverberant environment. Real-data experimental results indicate that satisfactory SSL performance is attained by the TR–SBL method. When the frequency of the sound source is low, a spatial resolution of 0.1 m can be obtained by this method. In the future, problems such as the localization performance over the number and positions of the microphones should be investigated.

## Figures and Tables

**Table 1:** RMSEs of three methods.

SNR/dB RMSE/m	TR	*L*_1_ Norm	TR–SBL
−30	0.75	0.58	0
−20	0.62	0.52	0
−15	0	0.55	0
−10	0	0.49	0

**Table 2:** Elapsed time required by different grid numbers.

Grid Time/s	TR	*L*_1_ Norm	TR–SBL
6 × 6	1.02	1.73	1.20
8 × 8	1.05	1.74	1.22
9 × 9	1.10	1.77	1.23
10 × 10	1.12	1.78	1.23
10 × 20	1.29	1.79	1.25
20 × 20	1.66	1.87	1.51

**Table 3:** RMSE of different methods under different bandwidths.

Bandwidth/Hz RMSE/m	TR	*L*_1_ Norm	TR–SBL
125–250	0.50	0.37	0.37
250–500	0.72	0.36	0.17
500–1000	0.59	0.38	0.10
125–1000	0.64	0.10	0.10

## Data Availability

The data that support the findings of this study are available from the corresponding author, upon reasonable request.
